# P04.80. Navigating the divide: women's engagement with conventional and complementary medicine in pregnancy

**DOI:** 10.1186/1472-6882-12-S1-P350

**Published:** 2012-06-12

**Authors:** A Steel, D Sibbritt, J Adams, J Daley

**Affiliations:** 1 University of Technology, Sydney, Brisbane, Australia; 2 University of Newcastle, Newcastle, Australia

## Purpose

Whilst it is reported that a significant percentage of women use complementary and alternative medicine (CAM) in pregnancy, little is known about the level at which they consult with CAM practitioners as part of their CAM use. Even less is known about the effect that the practice of consulting with CAM practitioners has on women’s patterns of use for conventional maternity care.

## Methods

This research was conducted as part of a sub-study of the Australian Longitudinal Study of Women’s Health (ALSWH) which investigated women’s use of health care during pregnancy and birthing, targeting women who had recently given birth (n=2445).

## Results

Of the women who responded (n=1835), almost all had consulted with a conventional care practitioner (99.8%), and just under half (49.4%) consulted with a CAM practitioner of some kind, most common being a massage therapist (34.1%), chiropractor (16.3%) and a meditation/yoga class (13.6%). The women consulted with these different CAM practitioners for a variety of reasons, such as a chiropractor for back pain (11.3%). There was also a high rate of self-prescription, particularly for vitamins (43.7%) and herbal teas (23.5%). Women were more likely to consult with CAM practitioners during pregnancy if they had private health insurance. In contrast, those consulting with CAM practitioners tended to consult less frequently with conventional practitioners.

## Conclusion

Most women wishing to access CAM during pregnancy create their own system of integrative health care to ensure they receive the type of care they feel they need by navigating between CAM and conventional care practitioners. Practitioners and policy makers need to further explore this under-researched area to enable the provision of adequate patient-centered care to women during pregnancy and birth.

